# Role of colonic microbiota in the pathogenesis of ulcerative colitis

**DOI:** 10.1186/s12876-019-0930-3

**Published:** 2019-01-14

**Authors:** Ling-yan Pei, Yu-shi Ke, Huan-hu Zhao, Lin Wang, Chao Jia, Wei-zhi Liu, Qian-hui Fu, Meng-ni Shi, Jian Cui, Shu-chun Li

**Affiliations:** 1 School of Pharmacy, Minzu University of China, 27 South Street, Zhongguancun, Beijing, 100081 China; 2 Key Laboratory of Ethnomedicine (Minzu University of China), Ministry of Education, Beijing, 100081 China; 3 Center for Drug Evaluation, China Food and Drug Administration, Beijing, 100081 China; 4 Department of Histology and Embryology, Xinxiang Medical University, Xinxiang, 453003 Henan China; 5 Department of Pathology, Beijing Children’s Hospital, Capital Medical University, National Center for Children’s Health, Beijing, 100045 China

**Keywords:** Ulcerative colitis, Intestinal microbiota, Microbiology

## Abstract

**Background:**

Recent studies have found gut microbiota to be closely associated with onset and perpetuation of UC. Currently, studies about gut microbiota have mainly covered samples collected from the intestinal lumen. However, the luminal flora is only part of the gut microbiota. Studies of the changes in mucosal flora under pathological conditions have been lacking. In this study, we investigated the correlation between the onset of UC and flora changes in different intestinal layers.

**Methods:**

The dextran sulfate sodium(DSS)-induced UC model was established by exposing mice to cycles of DSS. The luminal contents, an inner mucus layer, and outer mucus layer were harvested under sterile conditions. The samples were then analyzed using high-throughput sequencing of 16S rRNA V3 + V4 amplicons. The colonic microbiota composition and diversity were analyzed and compared using MetaStat, LefSe, multivariate analysis of variance, and spatial statistics.

**Results:**

The DSS-induced UC mouse model was successfully established. The diversity of the microbiota from luminal content, the outer mucus layer, and inner mucus layer were significantly different in both control and UC model groups. The statistically different OTUs belonged to Lachnospiraceae and Ruminococcaceae families within the order Clostridiales were mainly localized to the outer mucus layer.

**Conclusions:**

The alterations in flora composition and diversity mainly occurred in the colonic outer mucus layer. The change of flora in the colonic mucus layers is of great significance in the understanding of common features of gut flora in IBD and the understanding of the relationship between gut flora and disease progression.

**Electronic supplementary material:**

The online version of this article (10.1186/s12876-019-0930-3) contains supplementary material, which is available to authorized users.

## Background

Ulcerative colitis (UC) is a chronic, spontaneous inflammatory bowel disease affecting the innermost mucosa lining of the colon and rectum [1]. Extensive studies over the past decade have demonstrated that environmental factors, especially bacterial microflora, genetic and immunological factors play a substantial role in the pathogenesis of UC [2].

The dysbiosis of gut microbiota plays crucial roles in the pathogenesis of ulcerative colitis [3–5]. In mice with ulcerative colitis induced by DSS, the population of intestinal microflora, *Bacteroides distasonis, Clostridium spp*., and *Clostridium difficile*, increased significantly [6]. The relative shifts in abundant 16S rRNA (gene) phylotypes of *Akkermansia muciniphila* and *Enterobacteriaceae* are associated with the disease activity index of UC in DSS-induced UC mouse model. *Bifidobacterium* and the *Lactobacillus* group were increased in active IBD patients, while the population of butyric acid-producing bacteria decreased to some extent [7].

The intestinal epithelium and the tight junctions form a barrier that prevents permeation of pathogens from the luminal environment into the mucosal tissues and circulatory system. The integrity and normal regeneration capability of the mucosal surface epithelium are the structural basis of the intestinal mucosal barrier. The intestinal mucosal epithelium consists of absorptive enterocytes, goblet cells, and Paneth cells. Goblet cells secrete highly glycosylated gel-forming mucins that form a hydrophilic mucus on the epithelial layer. Due to the structural features and negative surface charge, mucin is able to trap and wrap up bacteria. The chemical groups exposed on mucin surface share similar structures with the intestinal epithelium, which facilitates the recognition and adhesion of bacteria. In addition, mucin binds to adhesion molecule on the intestinal epithelium, which can competitively inhibit the binding of harmful bacteria to adhesion molecules and subsequently inhibit the colonization of bacteria. The density of mucus is higher in the inner layer and decreases gradually toward the intestinal lumen. The outer mucus layer is loose and becomes habitat for commensal bacteria. If colonic epithelium is not covered by mucin, colitis will occur as a result of host immune response to invading bacteria [8]. Current studies of gut microbiota in UC use luminal content samples frequently. However, the luminal content flora can only partially reflect the alteration of gut microbiota. Research into the alternation of mucus layer flora under pathological conditions has been sparse. In this study, we established a DSS-induced mice UC model. Next, we conducted high throughput sequencing of the outer mucus layer flora, the inner mucus layer flora, and the luminal content flora. We then analyzed the differential counts of flora under pathological conditions in an attempt to understand the role of microbiota in the pathogenesis of UC.

## Methods

### Experimental animals and establishment of UC model

Thirty 9-week old male SPF C57BALB/c mice weighing 22 ± 2 g were purchased from Beijing Vital River Laboratory Animal Technology Co., Ltd. (Beijing, China; License #: SCXK (JING) 2012–0001). They were housed 3–5 mice per cage in an SPF animal facility of Institute of Science and Technology, National Health and Family Planning Commission of the People’s Republic of China (SYKX (JIGN) 2016–0010). The animals were randomly divided into a normal control group (*n* = 10) or UC model group (*n* = 20). A UC mouse model was established using the method reported by Benoit Chassaing [9]. Control mice received sterile drinking water throughout the study. UC model group mice were exposed to three cycles of DSS treatment. During the first cycle, animals were orally treated with 2.5% DSS (molecular weight 36–50kD; MP Biomedicals, US) for 7 days followed by 7 days of normal drinking water. During the second cycle, animals were orally treated with 2.0% DSS for 7 days followed by 7 days of normal drinking water. During the third cycle, animals were orally treated with 2.5% DSS for 2 days, then with 2.0% DSS for 5 days, followed by 2 days of normal drinking water. After the UC model was established, the animals were sacrificed by cervical dislocation and colons were excised and prepared for histopathological examination. The experimental protocols were performed after approval and in accordance with the guidelines set by the Ethical Committee of Minzu University of China (Protocol number: 201702).

### Isolation of intestinal content, an external mucus layer, and internal mucus layer

Intestinal segments were opened longitudinally. The contents were gently picked away using forceps until no visible particles remained. The intestinal content was placed in a 2 ml sterile cryopreservation tube and kept in liquid nitrogen. The outer mucus layer was gently scraped off, placed in 2 ml sterile cryopreservation tube and kept in liquid nitrogen. The rest of the colon tissue (inner mucus layer) was rinsed with PBS, minced, placed in a 2-ml sterile cryopreservation tube, and kept in liquid nitrogen.

### Extraction of total DNA

Genomic DNA of luminal content-, inner and outer mucosa-associated flora were extracted from using a fecal DNA extraction kit (Tiangen Biotech (Beijing) Co., Ltd.; product number: DP304).

### High-throughput sequencing of 16S rRNA amplicons

The purity and concentration of extracted genomic DNA were as determined using agarose gel electrophoresis. Then the genome DNA sample was diluted to 1 mg/μl with sterile water for PCR amplification. The primers were 341F: CCTAYGGGRBGCASCAG; and 806R: GGACTACNNGGGTATCTAAT. PCR reaction (30 μL) consisted of Phusion Master Mix (2×), 15 μl; each of the primer (2 μM), 3 μl; gDNA (1 ng/μL), 10 μL (5–10 ng); H_2_O, 2 μL. The thermal cycles included predenaturation at 98 °C for 1 min, and 30 cycles of 98 °C for 10s, 50 °C for 30s, 72 °C for 30s; and followed by a final extension at 72 °C for 5 min. PCR product was analyzed using 2% agarose gel electrophoresis. According to concentrations, the PCR products were mixed in the same concentration, then mixed with 1 × TAE buffer and purified using 2% agarose gel. PCR products with the size of 400–450 bp were recovered from the gel and further purified using GeneJet Gel Extraction Kit (Thermo Fisher Scientific). DNA libraries were generated using the NEB Next® Ultra™ DNA Library Prep Kit for Illumina (New England Biolabs, US) according to the manufacturer’s instructions. After Qubit quantification and quality control, these libraries were sequenced on the Illumina HiSeq system. We yielded 6,130,487 raw reads. After adaptor clipping, decloning, and rejecting low quality sequencing, 4,907,035 clean reads were obtained. Each read had Q20 > 98% and Q30 ≥ 97%.

### Bioinformation and biostatistics analysis

After trimming and quality filtering of the raw data, we obtained a clean dataset for construction of molecular operational taxonomic units (OTUs). Representative OTU was annotated to species, then the corresponding species information and species abundance information was delineated. Then the relative OTUs abundance, alpha diversity metrics, and Venn diagram were analyzed to obtain the information about bacterial species richness, proportional abundances of species (evenness), and OTUs differed or shared between groups. By constructing a phylogenetic tree using multiple sequence alignment of OTUs, we assessed microbial community structure differences between different samples and groups for analysis using ordination approaches, such as PCoA, PCA, or NMDS.

The microbiota composition and diversity were analyzed using MetaStat, LefSe, multivariate analysis of variance, and spatial statistics. Generalized linear mixed modeling was performed using the onset of UC as the dependent variable and repeat measurements of OTU abundances of luminal contents flora, inner mucus layer flora, and outer mucus layer flora as independent variables. The results showed that the distribution of 3862 OTUs in each layer was not repeated measurement data. Therefore, multiple logistic regression was used to analyze the relationship between UC onset and OTU abundance in each layer.

## Results

### Evaluation of disease model

**Fig. 1:**
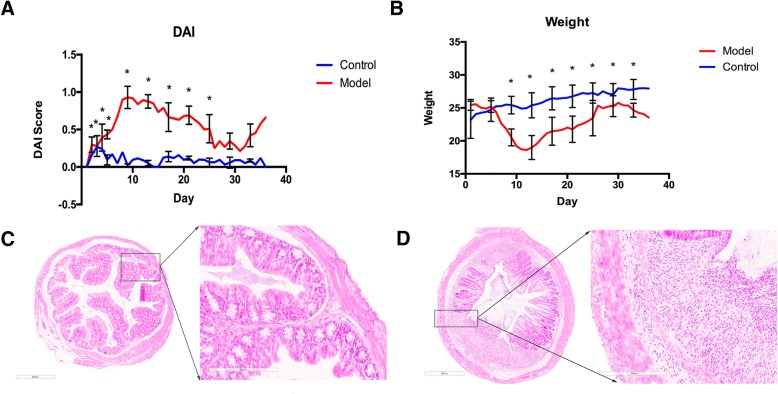
Evaluation of DSS-induced UC mice model. **a**. DAI of the control and model groups. **b**. Body weight of control and model group. **c**. Histological examination of colon tissue of control mice. The tissue sample was observed with 4.5-fold (left) and 20-fold (right) magnification. **d**. Histological examination of colon tissue of UC model mice. The tissue sample was observed with 4.5-fold (left) and 20-fold (right) magnification. Colonic biopsy sections from control and model groups were counterstained with hematoxylin. DAI: Disease active index; UC: Ulcerative colitis

The mice DAI index and body weight were analyzed using repeated measures ANOVA. The results showed that the DAI and body weight of model group differed significantly from the control group after time correction (*F = 168.66, P≦0.05; F = 10.881, P≦0.05*). During the first week of the model establishment, the model group was in the acute phase and DAI showed a significant increase. Then the DAI of the model group decreased to some extent but remained higher than that of the control group. During the last week of DSS exposure, the DAI of the model group increased again (Fig. 1a). The body weight of the UC model group was significantly lower than that of the control group throughout the modeling process (Fig. 1b). The histopathological slides were scanned using Leica APERIO AT2 and observed under 4.5-fold and 20-fold magnitude. The results showed that the colon tissue from the control group had a smooth surface, no erosion, ulcer, or crypt abscess (Fig. 1c). In contrast, the colon tissue from the UC model group had inflammatory infiltration in submucosa through the muscular layers of the colon wall, and necrosis of intestinal epithelium (Fig. 1d). These results demonstrated the UC mouse model was established successfully.

### Microbiota diversity analysis

**Fig. 2:**
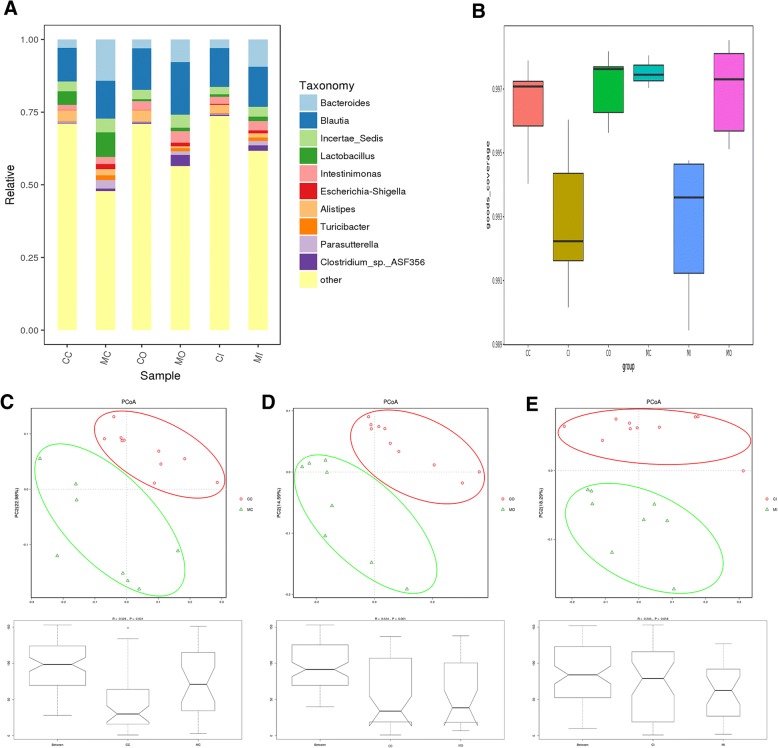
Diversity analysis of the microbiota. **a**. Comparison of the relative abundance of bacteria in three layers at genus level between groups. The microbial composition within the luminal content, inner layer and outer mucus layer of SPF mice was determined by 16S amplicon analysis. Representative bar graphs from control and model groups are shown. **b**. Comparison of alpha diversity index (good_coverage index) between groups. **c**. Comparison of luminal contents flora beta diversity index between groups (Unifrac weighted distance PCoA and Anosim analysis). **d**. Comparison of outer mucus layer flora beta diversity index between groups (Unifrac weighted distance PCoA and Anosim analysis). **e**. The comparison of inner mucus layer flora beta diversity index between groups (Unifrac weighted distance PCoA and Anosim analysis). Principle coordinates analysis (PCoA) on weighted UniFrac distances was performed on all operational taxonomic units. The closer the samples are to one another the more similar the bacterial diversity and richness are. *P*-values to determine the statistical significance of clustering were calculated using the Anosim method. *CC: the luminal content of control group. CO: the outer mucus layer of the control group. CI: the inner mucus layer of the control group. MC: the luminal content of the model group. MO: the outer mucus layer of the model group. MI: the inner mucus layer of the model group

At the genus level, ten bacteria including Bacteroides, Blautia, incertae_sedis, Lactobacillus, Intestinimonas, Escherichia-Shigella, Alistipes, Turicibacter, Parasutterella, and Clostridium_sp._ASF356 had high relative abundance in both control and model groups (Fig. 2a). We then conducted an alpha diversity comparison between different colon layers of control and UC model groups. We compared Chao1, observed_species, Shannon, and Simpson indices between the control and UC model groups. The results showed no significant difference regarding richness and diversity; while goods_coverage indices indicated significant differences between groups (Fig. 2b). In addition, none of the above-mentioned indices differed between the inner mucus layer flora and outer mucus layers flora between groups.

Principle coordinates analysis (PCoA) of the bacterial communities, including luminal contents, inner and outer mucus layers of control and UC group, derived from the weighted UniFrac distance matrix was conducted. The results showed that there was no overlap between samples from each group and the distance between groups was relatively large, which suggested that there were fewer shared OTUs common to both these groups and more different OTUs. Anosim analysis indicated that the inter-group difference was significantly greater than the intra-group difference (R > 0, *P* < 0.05) (Fig. 2c, d, and e). Adnois test derived from the weighted UniFrac distance matrix showed that the grouping factors contributed more to the OTUs differences observed in this study (*P* < 0.05) (Table 1).

**Table 1:** UniFrac weighted distance Adonis analysis between groups

Compare	F_value	R^2^	*P*_value
CC-MC	5.960654427	0.271424262	0.001
CI-MI	3.538972977	0.181123797	0.031
CO-MO	10.30440982	0.391736971	0.001

*CC: the luminal content of control group. CO: the outer mucus layer of control group. CI: the inner mucus layer of control group. MC: the luminal content of model group. MO: the outer mucus layer of model group. MI: the inner mucus layer of model group

### Analysis of differentially abundant flora

#### Differentially abundant flora in luminal contents, an inner mucus layer, and outer mucus layer

**Fig. 3:**
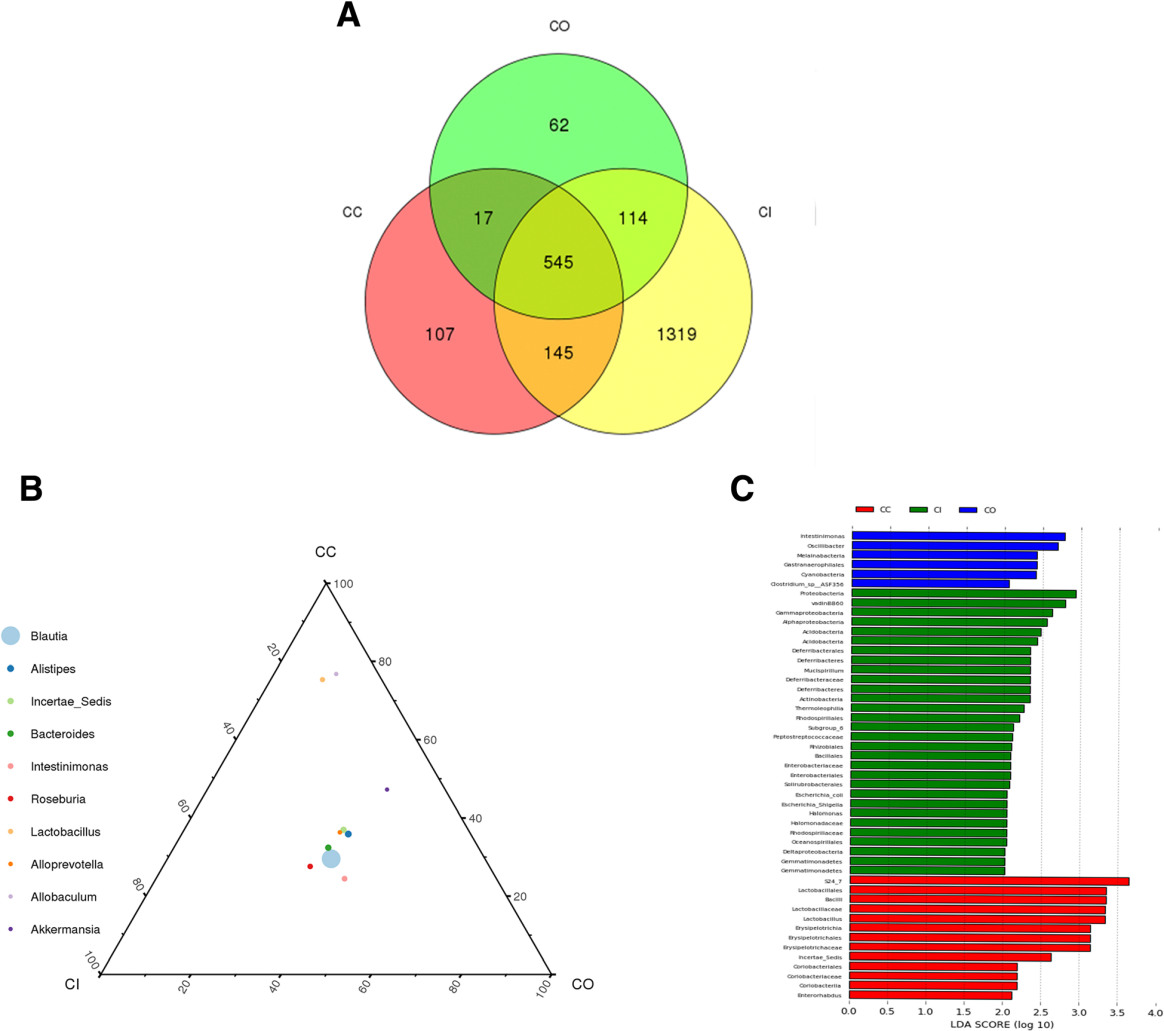
Differentially abundant bacteria in the luminal content flora, inner mucus layer flora, and outer mucus layer of the flora of the control group. **a**. Venn diagram showed differentially abundant bacteria in the luminal content flora, inner mucus layer flora, and outer mucus layer flora in the control group. **b**. Ternary diagram showed the differentially abundant bacteria in the luminal content flora, inner mucus layer flora, and outer mucus layer flora in the control group; **c**. The distribution histogram in LDA value showed the differentially abundant bacteria in the luminal content flora, inner mucus layer flora, and outer mucus layer flora of control group. LDA bar graphs of taxa/clades that are differentially abundant in the luminal content (red), inner layer (green) and outer mucus layer (blue) of the control group. Taxa in this graph were both statistically significant(*P* < 0.05) and had an LDA Score > 2, considered a significant effect size *CC: the luminal content of control group. CO: the outer mucus layer of the control group. CI: the inner mucus layer of the control group.

In control animals, a total of 545 OTUs were shared among the three layers. Luminal content-, outer mucus layer-, and inner mucosa layer-specific OTUs were 107, 62, and 1319, respectively (Fig. 3a). At the same taxonomy level, the abundance of the same species was different among different colon tissue layers. For example, at the genus level, the abundance of Bacteroides in each layer, ranked from highest to lowest, was CO > CI > CC; while that of Lactobacillus was CC > CI > CO (Fig. 3b). Forty-eight differentially abundant OTUs were found in three layers. Six OTUs, including Intestinimonas, Oscillibacter, and Melainabacteria, were enriched in the outer mucus layer (shown in blue color in Fig. 3c). Twenty-nine OTUs, including Proteobacteria, Gammaproteobacteria, and Alphaproteobacteria, were enriched in the inner mucus layer (shown in green in Fig. 3c). Thirteen OTUs, including Lactobacillales, Bacilli, and Lactobacillaceae, were enriched in luminal contents (shown in red in Fig. 3c).

**Fig. 4:**
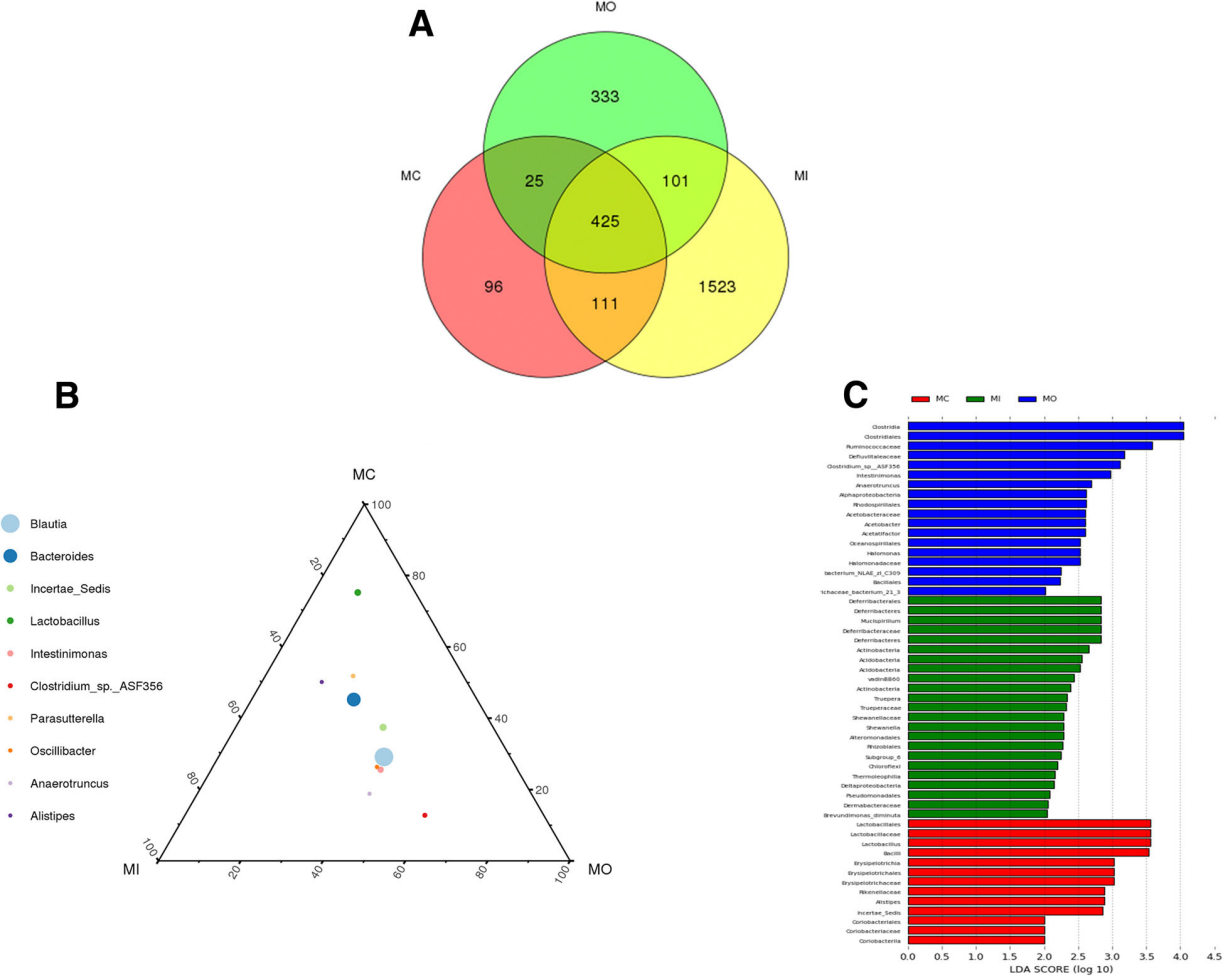
Differentially abundant bacteria in the luminal content flora, inner mucus layer flora, and outer mucus layer flora of UC model group. **a**. Venn diagram shows the differentially abundant bacteria in the luminal content flora, inner mucus layer flora, and outer mucus layer flora of UC model group; **b**. Ternary diagram showed the differentially abundant bacteria in the luminal content flora, inner mucus layer flora, and outer mucus layer flora of UC model group; **c**. The distribution histogram in LDA value showed the differentially abundant bacteria in the luminal content flora, inner mucus layer flora, and outer mucus layer flora of UC model group. LDA bar graphs of taxa/clades that are differentially abundant in the luminal content (red), inner layer (green) and outer mucus layer (blue) of the model group. Taxa in this graph were both statistically significant(*P* < 0.05) and had an LDA Score > 2, considered a significant effect size. *MC: the luminal content of the model group. MO: the outer mucus layer of the model group. MI: the inner mucus layer of the model group

In UC model animals, a total of 425 OTUs were shared among the three layers. Luminal contents-, outer mucus layer-, and inner mucosa layer-specific OTUs were 96, 333, and 1523, respectively (Fig. 4a). At the same taxonomy level, the abundance of same species differed across different colon tissue layers. For example, at the genus level, the abundance of Bacteroides in each layer, ranked from highest to lowest, was MC > MI > MO; while that of Lactobacillus was MC > MI > MO (Fig. 4b). Fifty-four differentially abundant OTUs were enriched from three layers. Eighteen OTUs, including Clostridia, Clostridiales, Ruminococcaceae, were enriched in the outer mucus layer (shown in blue color in Fig. 4c). Twenty-three OTUs, including Deferribacterales, Deferribacteres, Mucispirillum, were enriched in the inner mucus layer (shown in green color in Fig. 4c). Thirteen OTUs, including Lactobacillales, Lactobacillaceae, Lactobacillus, were enriched in luminal contents (shown in red in Fig. 4c).

#### Comparison of the differentially abundant flora of luminal contents, inner mucus layer and outer mucus layer between two groups

**Fig. 5:**
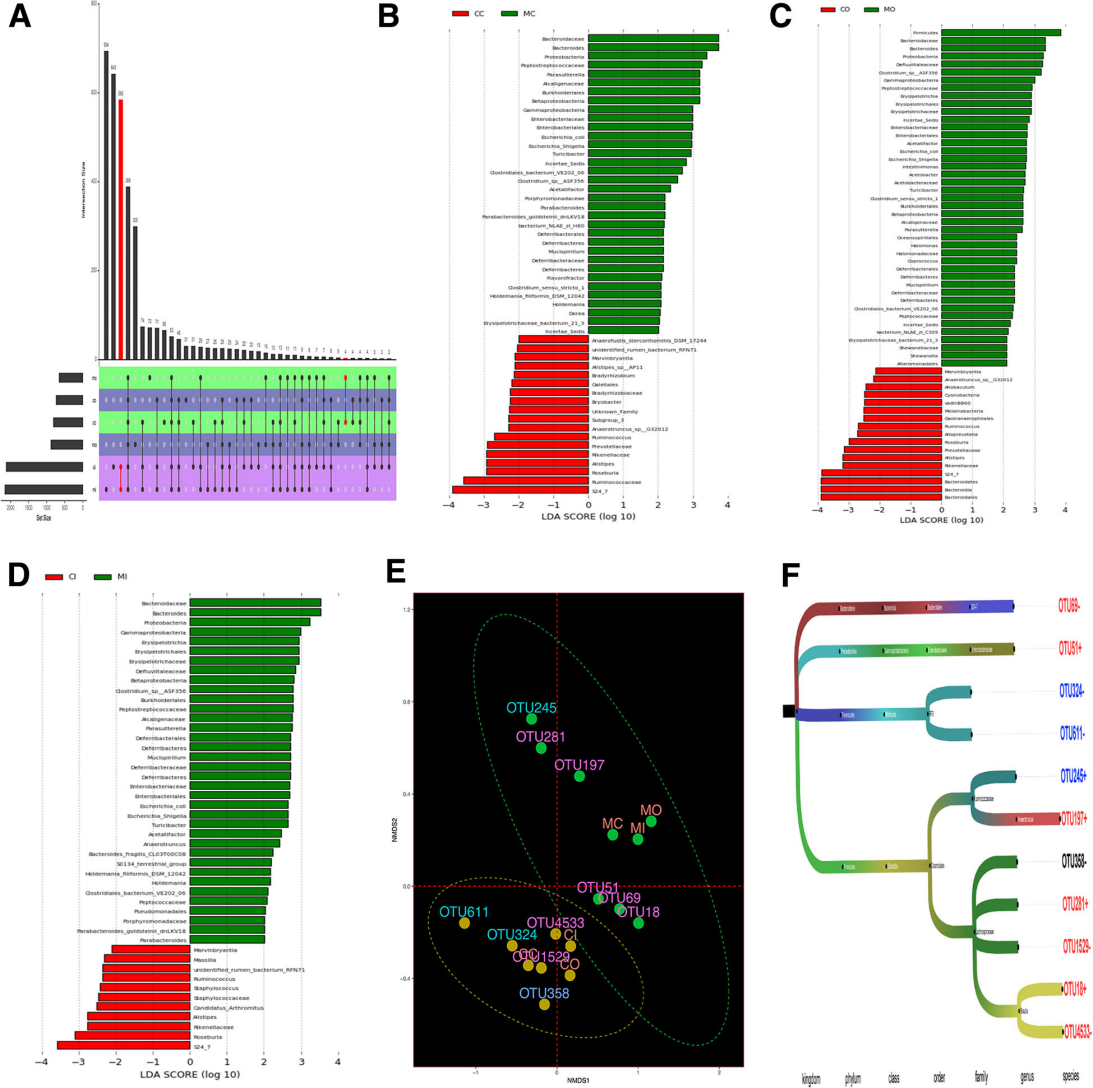
Differentially abundant bacteria among the flora of the luminal contents, an inner mucus layer, and outer mucus layer between control and UC model groups. **a**. The UPSET R analysis of the differentially abundant bacteria of luminal contents flora, inner mucus layer flora and outer mucus layer flora of control and model groups. **b**, **c**, **d**: From left to right, the distribution histogram in LDA value of luminal contents flora, inner mucus layer flora, and outer mucus layer flora, respectively. **e**. Multiple logistic regression analysis of the relationship between UC onset and the OTU abundance of each layer. **f**. The comparison of multiple logistic regression analysis of differentially abundant bacteria between two groups. *CC: the luminal content of control group. CO: the outer mucus layer of the control group. CI: the inner mucus layer of the control group. MC: the luminal content of the model group. MO: the outer mucus layer of the model group. MI: the inner mucus layer of the model group

Both control and model groups shared 491 luminal flora OTUs, 447 outer mucus layer flora OTUs, and 1361 inner mucus layer flora OTUs (Fig. 5a).

From luminal contents samples, 52 differentially abundant OTUs were identified from control and model groups; among which 18 (e.g., Marvinbryantia, Bradyrhizobium, Gaiellales) were enriched in the control group (shown in red) and 34 (e.g., Bacteroidaceae, Bacteroides, Proteobacteria) were enriched in the model group (shown in green) (Fig. 5b). From the inner mucus layer, 47 differentially abundant OTUs were identified from control and model groups; among which 11 (e.g., Marvinbryantia, Massilia, Ruminococcus) were enriched in the control group (shown in red) and 36 (e.g., Bacteroidaceae, Bacteroides, Proteobacteria) in the model group (shown in green) (Fig. 5c). From the outer mucus layer, 60 differentially abundant OTUs were identified from control and model groups; among which 17 (e.g., Marvinbryantia, Allobaculum, Cyanobacteria) were enriched in the control group (shown in red) and 43 (e.g., Firmicutes, Bacteroidaceae, Bacteroides) in the model group (shown in green) (Fig. 5d).

The metastat analysis was performed to compare the flora relative abundance between samples. For luminal content flora and inner mucus layer flora, no bacteria showed differences in abundance between the control and model groups. For the outer mucus layer flora, the abundances of Peptostreptococcus, Alistipes, and Turicibacter were significantly different between the control and model groups [Additional file 1: Table S1, Additional file 2: Table S2, available as Supplementary data at *BMC Gastroenterology* online]. Multiple logistic regression analysis of the relationship between UC onset and OTU abundance in each layer showed that the following OTUs were significantly different between control and UC mice: OTU245, OTU324, OTU611 of luminal contents flora, OTU18, OTU51, OTU69, OTU4533, OTU197, OTU1529, and OTU281 of outer mucus layer flora and OTU358 of inner mucus layer flora. The outer mucus layer of flora had more OTUs that differed significantly between the control and UC groups (Table 2). The OTUs that indicated an increased risk for UC were OTU245, OTU18, OTU51, OTU197, and OTU281; while the OTUs that indicated a decreased risk for UC were OTU324, OTU611, OTU69, OTU4533, OTU1529, and OTU358 (Fig. 5e). The above-mentioned OTUs were mainly annotated to Lachnospiraceae and Ruminococcaceae in the order Clostridiales (Fig. 5f).

**Table 2:** Distribution of differentially abundant OTUs and their roles

OUT id	c-pvalue	o-pvalue	i-pvalue	Main action area	Risk of disease
OTU245	0.023	0.330	0.435	C	+
OTU324	0.038	0.414	0.604	C	–
OTU611	0.036	0.997	0.640	C	–
OTU18	0.146	0.048	0.629	O	+
OTU51	0.148	0.042	0.890	O	+
OTU69	0.195	0.046	0.626	O	–
OTU4533	0.304	0.026	0.570	O	–
OTU197	0.344	0.041	0.485	O	+
OTU1529	0.245	0.034	0.228	O	–
OTU281	0.996	0.043	0.631	O	+
OTU358	0.821	0.345	0.047	I	–

*C: the luminal content. O: the outer mucus layer. I: the inner mucus layer

## Discussion

Among various chemically induced colitis models, DSS-induced colitis model is widely used because of its simplicity and many similarities with human ulcerative colitis. DSS carries a highly negative charge contributed by sulfate groups, is toxic to the colonic epithelia, and induces erosions that ultimately compromise barrier integrity resulting in increased colonic epithelial permeability [6, 9]. Further, its anticoagulant property aggravates intestinal bleeding. For unknown reasons, DSS-induced extensive pathology is confined to the large intestine, specifically the distal colon where an enormous number of microorganisms live. The mechanism by which DSS passes through mucosal epithelial cells remains unclear, but a recent publication suggests that DSS induces colitis in mice by forming nano-lipocomplexes with medium-chain-length fatty acids in the colon [10].

In the large intestine (colon) the mucus is continuous and has two layers. Tight stacking of polymeric glycoproteins adheres firmly to the epithelium forms a compact inner layer that is largely sterile [11]. Following proteolytic dispersion of mucin polymers, the outer layer is looser and contains commensal microbiota [12]. Only some bacterial species have a sufficient repertoire of genome-encoded catabolic glycosidic enzymes to break down complex mucus glycans, while most bacteria are asaccharolytic and use mucus glycoprotein as a source of carbon [13]. For these mucolytic bacteria, mucus is a potentially distinct microbiological niche based on the available carbon source [14]. The inner mucus layer is dense and impenetrable to bacteria, which allows it to protect the colonic epithelium. However, many microbial metabolites can enter the host, including short chain fatty acids that feed host epithelial cells and regulate immunity [15, 16]. This suggests that the bacteria residing in the colonic mucus microbiota niche contribute to the protective effects of the mucus barrier [17]. Under normal conditions, intestinal flora does not trigger inflammation at colonic mucosa. When the host is under stress or under some pathological conditions, the balance is perturbed, and harmful bacteria in the intestine or opportunistic pathogens may colonize another niche, invade the intestinal epithelium, and trigger inflammation. The tight junction proteins joining adjacent enterocytes are crucial for the maintenance of epithelial barrier integrity. During inflammatory processes, the tight junction proteins can be disrupted, allowing free passage of luminal contents into the lamina propria [18, 19].

The colonic mucus layers consist of polymeric sheets of Muc2 mucin and other bioactive molecules synthesized and secreted by goblet cells. Colonic commensal bacteria do not trigger excessive host immune responses. Colonic inner mucus layer separate intestinal epithelial cells from most of the bacteria, but the inner mucus layer is not sterile. The inner mucus layer comes into contact with a small number of bacteria. However, long-term exposure to bacteria can trigger a severe immune response; this immune response can affect the secretion of MUC2 mucin and the characteristics and function of the inner mucus layer [20]. In the case of ulcerative colitis, the thickness of the colonic mucus layer will decrease. In this way, bacteria can penetrate the inner mucus layer, reach the epithelium, and trigger severe colonic immune response. The dysbiosis of gut flora, together with an impaired intestinal clearance of bacteria, enhances the invasiveness of pathogens, disrupts the intestinal immune response, accelerates the intestinal inflammatory response and eventually leads to ulcerative colitis [21, 22].

The imbalance between beneficial and potentially harmful gut bacteria induces UC. However, studies of the locations of such dysbiosis have been lacking. In this study, we found that, in the control group, the luminal flora differed greatly from the inner mucus flora. In the UC model group, the difference between the inner mucus layer flora and the outer mucus layer flora and the difference between luminal flora and inner mucus flora were significant. These results indicated that the microbiota community structures of the inner and outer mucus layers were different in UC model mice. The different microbiota composition in inner and outer mucus layers may contribute greatly to the onset of UC. The luminal and outer layer flora were always in a dynamic state of flux. The dysbiosis of the inner and outer mucus layers is closely associated with the onset of UC. We also found that the flora of the outer mucus layers of control animals differed greatly from those of UC animals. However, the inner mucus layer flora and luminal flora did not differ between normal and UC animals. In addition, more bacteria in the outer mucus layer showed differences in abundance and structure relative to the inner mucus layer. Similar results were obtained from logistic regression analysis of the relationship between UC and OTU of different layers. These results indicated that dysbiosis occurred mainly in the outer mucus layer in UC animals and the mucus microbes are very active and competitive between different species. This may be due to the physiological functions of the outer mucus layer. The outer mucus layer is a very challenging habitat as it is undergoing rapid turnover with a time frame of several hours. As a result, microbes need to be fit enough to be renewed at the same rate while competing with each other for resources to persist in this stressful niche [14]. When the symbiotic niche is perturbed, some microbes will rapidly colonize and become dominant. The colonized microbes will reside in the outer mucus layer, produce protease, and degrade the MUC2 polymer [23], which leads to the invasion and direct contact of microbes with colonic epithelium. According to Venn diagram and MetaStat analysis, we found that the microbiota composition changed in UC mice and the flora change in the luminal sample could not reflect the flora change in the entire intestinal ecosystem. The flora of the mucus layers needs to be investigated as well. The luminal content sample has been used for intestinal microbiota analysis because it is less likely to be contaminated by alien species and is easy to collect. However, research into the correlation between dysbiosis and diseases shows that mucus flora usually displays significant alterations in species richness while luminal content flora does not, which means that much less information is derived from luminal content flora than from mucus flora [24]. Our results are consistent with this report, and the flora of the outer layer of mucus changed more significantly than either inner layer flora or luminal flora. In this way, the dynamic changes of mucus flora are of great importance regarding the characteristic microbiota changes in IBD.

About 99% of the normal human gut microbiota comprises of four phyla, namely Firmicutes (including the majority of Clostridium XIX and IV groups), Bacteroidetes (account for 90% of gut microbiota), Proteobacteria and Actinobacteria [25]. However, the analysis of mucosally associated bacteria showed enrichment of Streptococcal and Lactobacillus spp. (Bacillus subgroup of Firmicutes) [26]. Investigations have shown a relative decrease in the bacterial phyla Firmicutes and Bacteroidete, while an increase in Proteobacteria and Actinobacteria were observed with mucosal inflammation [27]. The research into the abundance of these bacteria in each mucus layers has been lacking. In this study, we found new phyla Proteobacteria, Firmicutes, Fusobacteria, and Bacteroidetes invaded into the outer mucus layer of UC model mice. We also observed invasion of phyla Proteobacteria, Gemmatimonadetes, Chloroflexi, Firmicutes, Verrucomicrobia, Bacteroidetes, Nitrospirae, Thaumarchaeota, Planctomycetes, and Actinobacteria into the inner mucus layer in UC mice. The invasion of these microbes may explain the alteration in microbiota composition here observed in UC mice. Bacteria of the phyla Actinobacteria and Proteobacteria are the most abundant in human and mouse colonic mucosa [13, 28]. Bacteroidaceae and Prevotellaceae families of phylum Bacteroidetes are abundant in mouse colonic mucosa. Our study showed similar results. Bacteroides species can break down a broad array of dietary polysaccharides and thus occupy a metabolic niche in which a variety of polysaccharides exist as sources of carbon [29]. When large numbers of glycanphiles, such as Bacteroides species, invade the inner mucus layer [30], they degrade glycan and spread with mucus. Because of gradual degradation, the inner mucus layer becomes thinner or even develops exposed patches. Then more bacteria or other microbes penetrate the inner mucus layer and adhere to the intestinal epithelial cells. When the bacterial metabolites are recognized by host intestinal epithelial cells and the immune system, a cascade of host immune responses will be triggered [26].

## Conclusions

In summary, we here found that the microbiota composition and changes in abundance took place mainly in the outer mucus layer of DSS-induced UC mice. The luminal flora did not change significantly. Therefore, the changes in flora in the colonic mucus layers is of great significance in the understanding of common features of gut flora in IBD and understanding of the relationship between gut flora and disease progression.

## Additional files

Additional file 1: CO-MO (Genus level) Metastat analysis. (PDF 68 kb)

Additional file 2: CO-MO (Species level) Metastat analysis. (PDF 42 kb)

## Abbreviations

16SrRNA: 16S ribosomal RNA
ANOVA: Analysis of Variance
CC: The luminal content of control group
CI: The inner mucus layer of control group
CO: The outer mucus layer of control group
DAI: Disease active index
DSS: Dextran sodium sulphate
IBD: Inflammation browel disease
IFN: Interferon
IL: Interleukin
LDA: Linear Discriminant Analysis
LefSe: LDA Effect Size
MC: The luminal content of model group
MI: The inner mucus layer of model group
MO: The outer mucus layer of model group
MUC2: Mucin2
NMDS: Non-metric multidimentional scaling
OTU: Operational taxonomic unit
PBS: Phosphate buffer saline
PCA: Principal component analysis
PCoA: Principal Co-ordinates analysis
PCR: Polymerase Chain Reaction
TLR: Toll-like receptor
UC: Ulcerative colitis
