# Supplementary material for: Role of colonic microbiota in the pathogenesis of ulcerative colitis
Source: BMC Gastroenterol. 2019 Jan 14;19:10. doi: 10.1186/s12876-019-0930-3 (PMC6332670; doi:10.1186/s12876-019-0930-3)
Supplement: Supplementary file 1 — CO-MO (Genus level) Metastat analysis. (PDF 68 kb) [file 12876_2019_930_MOESM1_ESM.pdf]

Supplementary file 1. CO-MO(Genus level) Metastat analysis

Taxa	mean.CO.	variance.CO.	standard.error.CO.	mean.MO.	variance.MO.	standard.error.MO.	p.value	q.value
k__Bacteria;p__Bacteroidetes;c__Bacteroidia;o__Bacteroidales;f__Rikenellaceae;g__Alistipes	0.0921799097228342	0.000821157249999059	0.00906177272943357	0.010745502167 2486	0.000109719987929871	0.0037033766337 2683	0.000999000999000999	0.0425833361015169
k__Bacteria;p__Firmicutes;c__Erysipelotrichia;o__Erysipelotrichales;f__Erysipelotrichaceae;g__Turicibacter	0.00252876245482506	2.2831306343540 5e-06	0.000477821162607314	0.0184223815789401	0.00015207938607379	0.0043600370708543	0.000999000999000999	0.0425833361015169
k__Bacteria;p__Bacteroidetes;c__Bacteroidia;o__Bacteroidales;f__Prevotellaceae;g__Alloprevotella	0.0400866833364502	0.000509583522418577	0.0071385119066 8319	0.0087608909046 3499	5.27191165062336e-05	0.0025670780204 8929	0.000999000999000999	0.0425833361015169
k__Bacteria;p__Firmicutes;c__Clostridia;o__Clostridiales;f__Lachnospiraceae;g__Acetatifactor	0.0033339602615 5412	6.7924642844936 7e-06	0.000824164078596833	0.0238188419094337	0.000120580845713174	0.0038823453883 1191	0.000999000999000999	0.0425833361015169
k__Bacteria;p__Firmicutes;c__Clostridia;o__Clostridiales;f__Lachnospiraceae;g__Roseburia	0.0531722121966488	0.000774204258637177	0.0087988877628 7763	0.0043013939520 2664	2.8990341969338 4e-05	0.0019036262096 7649	0.000999000999000999	0.0425833361015169
k__Bacteria;p__Firmicutes;c__Clostridia;o__Clostridiales;f__Ruminococcaceae;g__Ruminococcus	0.02611411021 1373	0.000297308613905474	0.0054526013416 1185	0.001552696955415	3.5642731812703 4e-06	0.000667483443733845	0.000999000999000999	0.0425833361015169
k__Bacteria;p__Proteobacteria;c__Alphaproteobacteria;o__Rhodospirillales;f__Acetobacterac	0	0	0	0.014366222642	0.000427803174	0.007312687386	0.000999000999	0.0425833361015

eae;g__Acetobacter				0816	506014	53936	000999	169	
k__Bacteria;p__Firmicutes;c__Clostridia;o__Clostridiales;f__Lachnospiraceae;g__Dorea	0.000145529217880315	1.329311765121 3e-08	3.645972799022 65e-05	0.00121183192768812	1.045650741344 21e-06	0.000361533321656562	0.000999000999000999	0.0425833361015169	
k__Bacteria;p__Firmicutes;c__Clostridia;o__Clostridiales;f__Lachnospiraceae;g__Marvinbryantia	0.00651769774479074	1.895860820933 91e-05	0.00137690261853695	0.000286285860813824	1.446553101509 55e-07	0.000134469006722253	0.000999000999000999	0.0425833361015169	
k__Bacteria;p__Proteobacteria;c__Alphaproteobacteria;o__Rhodospirillales;f__Rhodospirillaceae;g__Thalassospira	0.00293271029635925	3.533864497986 09e-06	0.000594463161010511	0.000198945521794757	1.595015718797 71e-07	0.000141200908 22998	0.000999000999000999	0.0425833361015169	
k__Bacteria;p__Firmicutes;c__Erysipelotrichia;o__Erysipelotrichales;f__Erysipelotrichaceae;g__Holdemania	2.118416000202 73e-05	2.039195406913 56e-09	1.428003994011 77e-05	0.000395704073165591	1.532145291341 99e-07	0.000138390086862372	0.000999000999000999	0.0425833361015169	
k__Bacteria;p__Firmicutes;c__Bacilli;o__Bacillales;f__Paenibacillaceae;g__Aneurinibacillus	0	0	0	0.00164998145058877	5.536319543393 33e-06	0.000831889381422895	0.000999000999000999	0.0425833361015169	
k__Bacteria;p__Firmicutes;c__Clostridia;o__Clostridiales;f__Caldicoprobacteraceae;g__Caldicoprobacter	0	0	0	0.00132516806512972	3.281976986782 36e-06	0.000640505365588607	0.000999000999000999	0.0425833361015169	
k__Bacteria;p__Firmicutes;c__Clostridia;o__Clostridiales;f__Syntrophomonadaceae;g__Dethiobacter	0	0	0	0.000670187236908555	8.545721020469 12e-07	0.000326835605092016	0.000999000999000999	0.0425833361015169	
k__Bacteria;p__Firmicutes;c__Clostridia;o__Clostridiales;f__Gracilibacteraceae;g__Gracilibacter	0	0	0	0.000673114589106748	9.043647191847 22e-07	0.000336222530324918	0.000999000999000999	0.0425833361015169	

k__Bacteria;p__Firmicutes;c__Clostridia;o__Clostridiales;f__Clostridiaceae_1;g__Candidatus_Arthromitus	0.000484435414541867	1.49962330968865e-07	0.000122459107855996	1.33926179889645e-05	1.43489773278668e-09	1.33926179889645e-05	0.000999000999000999	0.0425833361015169
k__Bacteria;p__Firmicutes;c__Clostridia;o__Clostridiales;f__Family_XI;g__Sedimentibacter	0	0	0	0.000563334213836161	6.06196196691393e-07	0.000275271728636313	0.000999000999000999	0.0425833361015169
k__Bacteria;p__Firmicutes;c__Clostridia;o__Clostridiales;f__Family_XIV;g__Anaerobranca	0	0	0	0.000478347727755804	7.30064719263681e-07	0.000302089539554021	0.000999000999000999	0.0425833361015169
k__Bacteria;p__Firmicutes;c__Clostridia;o__Clostridiales;f__Syntrophomonadaceae;g__Syntrophomonas	0	0	0	0.000319444891001596	2.15526335234987e-07	0.0001641365038752	0.000999000999000999	0.0425833361015169
k__Bacteria;p__Firmicutes;c__Clostridia;o__Clostridiales;f__Ruminococcaceae;s__bacterium_enrichment_culture_clone_BBMC-9	0	0	0	0.000272857184206236	1.45408471400432e-07	0.000134818614905561	0.000999000999000999	0.0425833361015169
k__Bacteria;p__Proteobacteria;c__Betaproteobacteria;o__Burkholderiales;f__Comamonadaceae;g__Delftia	0	0	0	6.26532992911205e-05	5.21842812206452e-09	2.55402332655374e-05	0.000999000999000999	0.0425833361015169
k__Bacteria;p__Actinobacteria;c__Actinobacteria;o__Bifidobacteriales;f__Bifidobacteriaceae;g__Gardnerella	0	0	0	0.000363808409212514	7.55959921473026e-07	0.000307400374404665	0.000999000999000999	0.0425833361015169
k__Bacteria;p__Firmicutes;c__Clostridia;o__Clostridiales;f__Peptococcaceae;g__Dehalobacter	0	0	0	0.00015741293950675	6.81841188847031e-08	9.23201758045764e-05	0.000999000999000999	0.0425833361015169
k__Bacteria;p__Proteobacteria;c__Deltaproteo	0	0	0	8.46196	1.31004	4.04667	0.00099	0.042583

bacteria;o__Desulfovibrionales;f__Desulfovibrionaceae;g__Desulfovibrio				61851484e-05	551237121e-08	380754121e-05	9000999000999	3361015169
k__Bacteria;p__Firmicutes;c__Clostridia;o__Clostridiales;f__Lachnospiraceae;g__Cellulosilyticum	0	0	0	0.000190856790151943	7.08457393995391e-08	9.41048214755354e-05	0.000999000999000999	0.0425833361015169
k__Bacteria;p__Firmicutes;c__Clostridia;o__Clostridiales;f__Clostridiaceae_1;g__Clostridium_sensu_stricto_12	0	0	0	0.000103720737370813	2.41899184259488e-08	5.49885424724424e-05	0.000999000999000999	0.0425833361015169
k__Bacteria;p__Firmicutes;c__Clostridia;o__Clostridiales;f__Ruminococcaceae;g__Acetivibrio	0	0	0	0.000124791582980224	3.83223315459518e-08	6.92119313647869e-05	0.000999000999000999	0.0425833361015169
k__Bacteria;p__Firmicutes;c__Bacilli;o__Lactobacillales;f__Streptococcaceae;g__Lactococcus	0	0	0	0.000193829967594806	1.43655696115549e-07	0.000134003589558055	0.000999000999000999	0.0425833361015169
k__Bacteria;p__Firmicutes;c__Bacilli;o__Bacillales;f__Pasteuriaceae;g__Pasteuria	0	0	0	6.34362591663683e-05	1.02327425136539e-08	3.57644070859108e-05	0.000999000999000999	0.0425833361015169
k__Bacteria;p__Actinobacteria;c__Coriobacteriia;o__Coriobacteriales;f__Coriobacteriaceae;g__Atopobium	0	0	0	6.13118404681605e-05	7.8622191290519e-09	3.13492805520555e-05	0.000999000999000999	0.0425833361015169
k__Bacteria;p__Firmicutes;c__Clostridia;o__Thermoanaerobacterales;f__Thermoanaerobacteraceae;g__Gelria	0	0	0	8.30456934333915e-05	1.50022429015259e-08	4.33045074177128e-05	0.000999000999000999	0.0425833361015169
k__Bacteria;p__Firmicutes;c__Clostridia;o__Clostridiales;f__Peptococcaceae;g__Desulfitobact	0	0	0	3.641739925654	3.845988534524	2.192597926696	0.000999000999	0.0425833361015

erium				65e-05	21e-09	84e-05	000999	169
k__Bacteria;p__Firmicutes;c__Clostridia;o__Clostridiales;f__Peptococcaceae;g__Cryptanaerobacter	0	0	0	6.38726482198432e-05	9.18448824019876e-09	3.38830493023406e-05	0.000999000999000999	0.0425833361015169
k__Bacteria;p__Firmicutes;c__Clostridia;o__Clostridiales;f__Clostridiaceae_1;g__Clostridium_sensu_stricto_6	0	0	0	5.93154509822368e-05	1.00351263373011e-08	3.54173798037437e-05	0.000999000999000999	0.0425833361015169
k__Bacteria;p__Firmicutes;c__Clostridia;o__Clostridiales;f__Defluviitaleaceae;g__Defluviitalea	0	0	0	5.95793187635631e-05	6.77480570796449e-09	2.91006995361892e-05	0.000999000999000999	0.0425833361015169
k__Bacteria;p__Firmicutes;c__Clostridia;o__Clostridiales;f__Gracilibacteraceae;g__Lutispora	0	0	0	3.14542618018578e-05	3.08972844812071e-09	1.96523804159977e-05	0.000999000999000999	0.0425833361015169
k__Bacteria;p__Firmicutes;c__Clostridia;o__Clostridiales;f__Family_XI;g__Tissierella	0	0	0	4.30656703917588e-05	4.60262141168597e-09	2.39859891699456e-05	0.000999000999000999	0.0425833361015169
k__Bacteria;p__Firmicutes;c__Bacilli;o__Bacillales;f__Paenibacillaceae;g__Thermobacillus	0	0	0	6.64610088437091e-05	1.20209324024793e-08	3.87635982631891e-05	0.000999000999000999	0.0425833361015169
k__Bacteria;p__Firmicutes;c__Clostridia;o__Clostridiales;f__Clostridiaceae_2;g__Alkaliphilus	0	0	0	3.23574887452002e-05	3.59372369775305e-09	2.11947036360297e-05	0.000999000999000999	0.0425833361015169
k__Bacteria;p__Proteobacteria;c__Gammaproteobacteria;o__Chromatiales;f__Ectothiorhodospiraceae;g__Thioalkalispira	0	0	0	4.63238390140333e-05	8.37227118627001e-09	3.23501761708302e-05	0.000999000999000999	0.0425833361015169

k__Bacteria;p__Actinobacteria;c__Nitriliruptoria;o__Nitriliruptorales;f__Nitriliruptoraceae;g__Nitriliruptor	0	0	0	6.63489101049364e-05	3.07339858593288e-08	6.19818379238314e-05	0.00099900099900099	0.0425833361015169
k__Bacteria;p__Firmicutes;c__Clostridia;o__Clostridiales;f__Family_XI;g__Peptoniphilus	0	0	0	4.89008436926502e-05	5.70979707460099e-09	2.67156252841876e-05	0.00099900099900099	0.0425833361015169
k__Bacteria;p__Firmicutes;c__Clostridia;o__Clostridiales;f__Ruminococcaceae;s__bacterium_enrichment_culture_clone_LDC-11	0	0	0	3.12512916933167e-05	2.45367236854736e-09	1.75131106908059e-05	0.00099900099900099	0.0425833361015169
k__Bacteria;p__Firmicutes;c__Clostridia;o__Clostridiales;f__vadinBB60;g__Clostridiales_bacterium_JN18_A56_K	0	0	0	4.37050295537748e-05	4.85577946743674e-09	2.46368105368693e-05	0.00099900099900099	0.0425833361015169
k__Bacteria;p__Actinobacteria;c__Actinobacteria;o__Actinomycetales;f__Actinomycetaceae;g__Actinomyces	0	0	0	4.6870102559137e-05	1.46808786725893e-08	4.28381819650842e-05	0.00099900099900099	0.0425833361015169
k__Bacteria;p__Proteobacteria;c__Epsilonprote	0	0	0	5.22340	1.05484	3.63120	0.00099	0.042583

obacteria;o__Campylobacterales;f__Helicobacteraceae;g__Wolinella				187446848e-05	967479301e-08	103201581e-05	9000999000999	3361015169
k__Bacteria;p__Proteobacteria;c__Gammaproteobacteria;o__Enterobacteriales;f__Enterobacteriaceae;g__Morganella	0	0	0	3.70115259651332e-05	8.51763554094165e-09	3.26298091109603e-05	0.000999000999000999	0.0425833361015169
k__Bacteria;p__Proteobacteria;c__Gammaproteobacteria;o__Xanthomonadales;f__Nevskiaceae;g__Nevskia	0	0	0	3.83664007183964e-05	9.30227626656934e-09	3.40996265862424e-05	0.000999000999000999	0.0425833361015169
