# Supplementary material for: Role of colonic microbiota in the pathogenesis of ulcerative colitis
Source: BMC Gastroenterol. 2019 Jan 14;19:10. doi: 10.1186/s12876-019-0930-3 (PMC6332670; doi:10.1186/s12876-019-0930-3)
Supplement: Supplementary file 2 — CO-MO (Species level) Metastat analysis. (PDF 42 kb) [file 12876_2019_930_MOESM2_ESM.pdf]

Supplementary file 2. CO-MO (Species level) Metastat analysis

Taxa	mean.CO.	variance.CO.	standard.error.CO.	mean.MO.	variance.MO.	standard.error.MO.	p.value	q.value
k__Bacteria;p__Firmicutes;c__Clostridia;o__Clostridiales;f__Lachnospiraceae;g__Blautia;s__Clostridiales_bacterium_VE202-06	0.0297006558205442	0.000394932595437186	0.0062843662802003	0.133210231440598	0.00194141465748765	0.0155780882070284	0.000999000999000999	0.0350507948735694
k__Bacteria;p__Firmicutes;c__Erysipelotrichia;o__Erysipelotrichales;f__Erysipelotrichaceae;g__Incertae_Sedis;s__Erysipelotrichaceae_bacterium_21_3	0.00825906672071325	5.47059785957856e-05	0.00233893092236144	0.08623066324482	0.00100325826417881	0.011198539325392	0.000999000999000999	0.0350507948735694
k__Bacteria;p__Firmicutes;c__Clostridia;o__Clostridiales;f__Ruminococcaceae;g__Anaerotruncus;s__Anaerotruncus_sp._G32012	0.373302082711393	0.0120594424337851	0.0347267079260115	0.0254402509629312	0.00107694718874081	0.0116025169076628	0.000999000999000999	0.0350507948735694
k__Bacteria;p__Firmicutes;c__Bacilli;o__Lactobacillales;f__Lactobacillaceae;g__Lactobacillus;s__mouse_gut_metagenome	0.162046901982967	0.014745056612961	0.0383992924582746	0.04463450748912	0.00060341827698606 4	0.008684888290776	0.000999000999000999	0.0350507948735694
k__Bacteria;p__Firmicutes;c__Bacilli;o__Bacillales;f__Staphylococcaceae;g__Staphylococcus;s__Staphylococcus_sp._SV3	0.0013787245837214	5.42336950791425e-	0.0007364352997999	0.0003194477024526	4.44030884347641e-	0.0002355925731924	0.0014907850854524	0.0492286639369432

	2	06	39	29	07	82	8	
k__Bacteria;p__Firmicutes;c__Clostridia;o__Clostridiales;f__Clostridiaceae_1;g__Clostridium_sensu_stricto_1;s__Clostridium_butyricum	0	0	0	0.00472652840959669	6.05962788168386e-05	0.0027521872850707	0.000999000999000999	0.0350507948735694
k__Bacteria;p__Actinobacteria;c__Actinobacteria;o__Bifidobacteriales;f__Bifidobacteriaceae;g__Gardnerella;s__Gardnerella_vaginalis_0288E	0	0	0	0.0184339298455473	0.00251793959822628	0.0177409822100775	0.000999000999000999	0.0350507948735694
k__Bacteria;p__Firmicutes;c__Bacilli;o__Bacillales;f__Bacillaceae;g__Bacillus;s__Bacillus_coagulans	0	0	0	0.00228761464063366	1.16352855086545e-05	0.00120598950599987	0.000999000999000999	0.0350507948735694
k__Bacteria;p__Firmicutes;c__Bacilli;o__Lactobacillales;f__Lactobacillaceae;g__Lactobacillus;s__Lactobacillus_sp._EMML_3041	0	0	0	0.0018885411624956	9.47829883347759e-06	0.00108847937701396	0.000999000999000999	0.0350507948735694
k__Bacteria;p__Proteobacteria;c__Gammaproteobacteria;o__Pseudomonadales;f__Moraxellaceae;g__Acinetobacter;s__Acinetobacter_calcoaceticus	0	0	0	0.00493864827254211	5.8500757666832e-05	0.00270418096812214	0.000999000999000999	0.0350507948735694
k__Bacteria;p__Bacteroidetes;c__Bacteroidia;o__Bacteroidales;f__Prevotellaceae;g__Prevotella;s__Prevotella_bivia	0	0	0	0.00390661308338855	8.10577431425383e-05	0.00318311449571285	0.000999000999000999	0.0350507948735694

k__Bacteria;p__Firmicutes;c__Clostridia;o__Clostridiales;f__Clostridiaceae_1;g__Clostridium_sensu_stricto_6;s__Clostridium_sp._M2_40	0	0	0	0.000966907543817645	1.85439586195214e-06	0.000481455587509395	0.000999000999000999	0.0350507948735694
k__Bacteria;p__Firmicutes;c__Bacilli;o__Bacillales;f__Paenibacillaceae;g__Thermobacillus;s__Thermobacillus_composti_KWC4	0	0	0	0.00112834221214777	4.47319922873044e-06	0.00074776326707894	0.000999000999000999	0.0350507948735694
k__Bacteria;p__Firmicutes;c__Bacilli;o__Lactobacillales;f__Lactobacillaceae;g__Lactobacillus;s__Lactobacillus_plantarum	0	0	0	0.00091882399788586	4.76948852880136e-06	0.00077213086073551 7	0.000999000999000999	0.0350507948735694
k__Bacteria;p__Proteobacteria;c__Epsilonproteobacteria;o__Campylobacterales;f__Helicobacteraceae;g__Wolinella;s__Wolinella_succinogenes	0	0	0	0.00051899148513859 4	8.37970769761856e-07	0.00032364540197603 9	0.000999000999000999	0.0350507948735694
k__Bacteria;p__Firmicutes;c__Bacilli;o__Bacillales;f__Paenibacillaceae;g__Paenibacillus;s__Bacillus_sp._DB1032010	0	0	0	0.00067014399545228 9	1.25868591857098e-06	0.00039665569430095 5	0.000999000999000999	0.0350507948735694
k__Bacteria;p__Proteobacteria;c__Gammaproteobacteria;o__Enterobacteriales;f__Enterobacteriaceae;g__Arsenophonus;s__secondary_endosymbiont_of_Bemisia_tabaci	0	0	0	0.00133333333333333	1.42222222222222e-05	0.00133333333333333	0.000999000999000999	0.0350507948735694
